# Keynote 3

**DOI:** 10.1093/eurpub/ckac092.003

**Published:** 2022-08-29

## Abstract

**Title of HEPA 2022 Keynote presentation:**

Are all physical activity health-enhancing? On the Physical Activity Paradox.

**Description of HEPA 2022 Keynote presentation:**

We know that physical activity is among the best investments for well-being and health. It’s beyond doubt that physical activity during leisure time promotes health. But, how can it then be that blue collar workers being physically active for several hours each working day—often walking more than 10 000 steps during working hours alone—have such poor fitness and health? Yes indeed, blue collar workers often have less beneficial lifestyle factors, but can it explain it all? Can it be that the physical activity performed as a productive work demand during working hours not provides the same health benefits as when performed during leisure time, termed the “Physical Activity Paradox”?

In my talk, I will present the background, findings and explanations for the Physical Activity Paradox. I will discuss if physical activity during productive work can be designed so workers get fit and healthy by performing their job (defined as the Goldilocks Work Principle). I will address the need for improving collaboration, common understanding and recommendations regarding physical activity at work and leisure. I will end up with, the need for a systemic population-based approach integrating work and leisure physical activity for particularly reaching blue-collar lower-socioeconomic groups.

